# Emotional Contagion: A Brief Overview and Future Directions

**DOI:** 10.3389/fpsyg.2021.712606

**Published:** 2021-07-16

**Authors:** Carolina Herrando, Efthymios Constantinides

**Affiliations:** Faculty of Behavioral, Management and Social Sciences (BMS), Department High-Tech Business and Entrepreneurship (HBE/ETM), University of Twente, Enschede, Netherlands

**Keywords:** emotions, emotional contagion, arousal, physiological reaction, emotional state, neuroscience, facial expression, decision-making process

## Abstract

Social interactions can trigger emotional contagion between individuals resulting in behavioral synchrony. Emotional contagion can be a very effective and attractive strategy in communication and advertising, and understanding the mechanisms underlying emotional contagion can help marketers to improve their commercial approaches or develop better ones. The purpose of this study is to review and classify the various methodologies and theoretical approaches on emotional contagion, identify the best practices in this domain, and identify ways of gaging and measuring emotional contagion. The study is based on a mini literature review. We identify different mechanisms and approaches to emotional contagion described in the literature. Emotional contagion can be triggered by facial expressions, indirect human interactions, and/or by observing other people's behavior in direct and indirect interactions. Furthermore, emotional contagion can be triggered physiologically or neurologically by synchronizing with the emotional state of others during human interactions. Regarding the assessment and measurement of emotional contagion, we argue that methods based on neuroscience tools are much more accurate and effective than methods based on traditional research approaches. The study identifies guidelines for research on commercial communication through emotional contagion that can be especially interesting for academia and marketing practitioners. The findings are important for field marketers interested in developing new individualized approaches in their commercial strategies and marketing in general. In addition, the study can become the basis of research that further refines and compares the efficacy of the various techniques and tools involved.

## Introduction

Emotion theorists have been describing emotions from a different level of complexity point of view (Darwin, 1872/1965; Izard, 1971, 1992; Ekman et al., 1983; Fischer et al., 1990; Ortony and Turner, 1990; Panksepp, 2004). With basic description, emotions are considered simple feelings of positive or negative pleasure and arousal (Russell, 2003). With a more intricate description, emotions are considered as a complex and organized family under a meaningful hierarchy (Fischer et al., 1990). In any case, emotions are innate so during any social interaction there will be always some emotion exposure.

When someone smiles at us, the natural reaction is to smile back in order to align with the emotion of the other person (Barger and Grandey, 2006; Hennig-Thurau et al., 2006; Smith and Rose, 2020). Instinctively, humans tend to align with the emotional states they perceive during interactions (Ekman et al., 1983; Hatfield et al., 1994; Barger and Grandey, 2006; Hennig-Thurau et al., 2006; Papousek et al., 2011; Kramer et al., 2014; Dixon et al., 2017; Prochazkova and Kret, 2017). Evidence suggests that emotions can be contagious and cause mind and body arousal (Schachter and Singer, 1962). Emotional contagion can be reflected in showing a similar facial, vocal, or postural expression, as well as similar neurophysiological and neurological reactions (Hatfield et al., 1994) toward the interacting party. As a response to emotional contagion, individuals show behavioral, attentional, and emotional synchrony (Hatfield et al., 1992). The domain of marketing uses interactions with human agents and objects as a method of commercial communications. Therefore, the understanding of emotional contagion mechanisms is of great importance for marketing research and practice.

Emotions have awakened the interest of researchers for quite some time (Bagozzi et al., 1999; Kranzbühler et al., 2020). The study of emotional contagion has been the focus of various disciplines because different types of interactions, such as commercial transactions, team communication, and human–robot interactions, can transfer emotions (Li et al., 2017; Wergin et al., 2018; Chen et al., 2019; Kuang et al., 2019; Matsui and Yamada, 2019; Mindeguia et al., 2021). Marketing research on emotional contagion has focused on understanding how positive or negative emotions converge in positive or negative consumer behavior (Hennig-Thurau et al., 2006; Dellarocas et al., 2007; Cowley, 2014; Kramer et al., 2014; Fox et al., 2018). However, while marketing and psychology studies usually employ self-reported techniques to understand consumer behavior, the study of emotional contagion is quite suited to drawing on neurophysiological techniques to explain how emotions trigger mind and body arousal (Bell et al., 2018; Verhulst et al., 2019; Bettiga et al., 2020).

This study conducts an integrative review of literature related to emotional contagion to expand the topic's theoretical foundations and identify the main methodological approaches for the study of emotional contagion. The review concludes with a discussion of the most appropriate methodologies to advance research on emotional contagion, and identifies various topics of further research.

## Theoretical Overview on Emotional Contagion

Historically, emotional contagion has been directly related to the feelings of empathy and sympathy (see Hatfield et al., 1994; Decety and Ickles, 2009), stemming from the German term *Einfühlung* (Stein, 1917/1964). Broadly, empathy has been described as the ability to share the experience of others and it has been related to the activation of neural structures and physiological responses (Singer and Lamm, 2009). Hence, in the study of empathy, emotional contagion in social interactions seems to be a crucial characteristic for understanding emotional states (Harrison et al., 2006). Theories of empathy study how humans and animals react to affective states as a primary form of emotional contagion (see Decety and Ickles, 2009; De Waal, 2010; Panksepp, 2011). The Perception Action Model (Preston and De Waal, 2002) helps to explain different levels of empathy from an evolutionary point of view, distinguishing and interrelating between the concepts of empathy, sympathy and emotional contagion, as well as other related concepts as imitation, compassion and mimicry (see also Hatfield et al., 1994; Decety and Ickles, 2009; Singer and Lamm, 2009).

Emotional contagion has been widely seen as being closely associated with emotional arousal (Hatfield et al., 1994; Prochazkova and Kret, 2017), and much of the literature on emotional contagion is indeed grounded on the theory of arousal (Mehrabian and Russell, 1974; Mehrabian, 1980; Russell, 1980, 2003). Such studies have stated that emotions can be classified according to the level of pleasure (positive/negative) and the level of arousal (relax/activated) experienced by individuals; emotions are therefore a combination of feelings with arousal. The review of the literature points to other theories that can support the conceptual framing of emotional contagion. Stress theory (Lazarus, 1966), for instance, suggests that the level of stress is associated with similar types of emotions, such as anxiety. Flow theory (Csikszentmihalyi, 1975) refers to different states of mind, such as boredom, apathy, flow, and anxiety, which vary depending on the level of the individual's skills with respect to facing challenges. According to Bandura (1973), social learning theory suggests that people learn from their cognitive experiences after being exposed to the behavior of others. Social exchange theory (Emerson, 1976) indicates that people lean from the experiences of others in making up their minds. In addition, cognitive appraisal theory (Moors, 2009; Moors et al., 2013) suggests that the subconscious cognitive experience affects emotions. Van Kleef (2009) argued that, according to social information theory applied to information processing, people tend to interpret the emotions linked to content, which indirectly influences their behavior. In this line, the similarity-attraction paradigm (Kidwell et al., 2020) supports the argument that people process both information and emotions at the same time, being attracted by similarities.

## Methodological Approaches to Emotional Contagion

### Emotional Contagion in Facial Expression Reactions

During human interactions, people tend to align with the emotional state of the other person, in terms not only of emotionally empathizing with the other but also of mimicking facial expressions and coping with bodily changes (Hatfield et al., 1994; Hess and Fischer, 2014; Aldunate and González-Ibáñez, 2017; Norscia et al., 2020). Studying facial expressions has been one of the main methodological approaches used to study emotional contagion (see Singer et al., 2004; Manera et al., 2013; Kret, 2015; Aldunate and González-Ibáñez, 2017; Dixon et al., 2017; Prochazkova and Kret, 2017; Fox et al., 2018; Smith and Rose, 2020). Many researchers have underlined the salience of facial expressions for emotional contagion, determining that in face-to-face interactions emotions can be transferred from the observed to the observer (Gellhorn, 1964; Izard, 1971; Ekman et al., 1983). For instance, the perceived quality of service in an organization can be affected by smiling employees (Barger and Grandey, 2006). Moreover, in audio-visual computer-mediated communication, smiles and emotions can be contagious via facial mimicry (Mui et al., 2018). Nevertheless, the psychological state of the individual can affect the processing of emotional information; for example, people with depression fail to differentiate between positive or non-emotional content (Goodin et al., 2019). However, a recent study showed that negative affective states have a stronger impact on negative emotions than on positive ones (Pinilla et al., 2020). In this vein, Deng and Hu (2018) assessed two types of contagion: mimicry-based for underlying positive emotional processing, and social appraisal for negative emotions. This study indicates that in emotional processing social contagion should be considered to be equally, or more, important than facial contagion. Nevertheless, it has been claimed that emotional mimicry depends on the social context (Hess and Fischer, 2014). In recent years, research on face-to-face contagion has gone beyond facial mimicry, focusing on how nonverbal cues can also transfer emotions; for example, through emotions (Aldunate and González-Ibáñez, 2017) or social media posts (Kramer et al., 2014; Pera, 2018). Facial expressions tend to be mimicked in face-to-face interactions; however, with the inclusion of online interactions, there is a need for further research on emotional contagion to uncover nonfacial interaction reactions. It results particularly interesting to deepen the research in nonfacial emotional contagion through the use of emojis or emoticons (Aldunate and González-Ibáñez, 2017; Smith and Rose, 2020).

### Emotional Contagion in Behavioral Reactions

Previous studies on consumer behavior have aimed to explain the role of emotions during the decision-making process (Hennig-Thurau et al., 2006; Felbermayr and Nanopoulos, 2016; Zablocki et al., 2019). Using traditional behavioral measures, researchers have concluded that emotions, whether positive or negative, can be contagious by observing other people's experiences (Cowley, 2014; Kramer et al., 2014). Kramer et al. (2014) argued that emotional contagion can occur even without face-to-face interaction; for example, during interactions on social media. Consequently, reading reviews and observing other people's behavior on social networks have been proven to trigger emotional contagion. In a social media experiment, Wakefield and Wakefield (2018) found that when people read negative product reviews or customer experiences they tend to feel greater anxiety. Since such content is often shared, the emotional valence of the information shared via social media is affected by the emotional content observed (Ferrara and Yang, 2015; Sciara et al., 2021). These studies have confirmed that even in the complete absence of nonverbal cues, emotions can be contagious. In fact, in online interactions facial mimicry does not seem to be crucial for emotional contagion. When interacting online, people associate positive reviews or high ratings with positive emotions, while low ratings and negative reviews are associated with negative emotions (Lindquist et al., 2016; Septianto et al., 2020; Xu et al., 2020). Therefore, emotions shared by others influence customer experience, which results in a specific behavioral response. For instance, positive emotional contagion leads to positive affect, satisfaction, and loyalty intentions (Hennig-Thurau et al., 2006); on the contrary, negative emotions discourage against making purchases (Dellarocas et al., 2007). Therefore, greater understanding of consumers' emotional and behavioral synchrony is of great importance for business and academia.

### Emotional Contagion in Physiological Reactions

Over the years, social neuroscientists have provided evidence that, during social interactions, the observation of another person's emotional state automatically activates the same autonomic nervous system response and neural representation of the affective state as that of the observer (Hatfield et al., 1994; Keysers and Gazzola, 2010; Anders et al., 2011; Eerola et al., 2016; Prochazkova and Kret, 2017). Early studies confirmed that the patterns of basic emotions can be physiologically identified and linked to arousal variations (Cannon, 1915; Ax, 1953; Schachter and Singer, 1962). In emotional contagion, positive and negative emotions are associated with physiological reactions (see reviews by Kreibig, 2010; Caruelle et al., 2019). Hence, emotional contagion during social interactions awakens affective arousal and cognition (Shamay-Tsoory, 2009; Kret, 2015).

The review of the literature on emotional contagion in physiological reactions reveals that the skin conductance response is a widely used method to understand how peoples' galvanic skin response varies depending on their level of arousal (Hatfield et al., 1994; Kreibig, 2010; Caruelle et al., 2019; Verhulst et al., 2019). This approach considers physiological arousal as an index to monitor anxiety (Hatfield et al., 1994). Therefore, different levels of arousal can be associated with several emotions (Peifer et al., 2014; Tozman et al., 2015; Fox et al., 2018). For example, anger and fear are linked to certain patterns of physiological arousal (James, 1890/1984; Ax, 1953). Particularly, high levels of arousal are associated with anxiety (Hatfield et al., 1994; Kreibig, 2010). However, it has been noted that physiological arousal is associated with the intensity (relax-activated) rather than the valence (positive/negative) of the emotion (Dysinger, 1931; McCurdy, 1950; Hatfield et al., 1994). Therefore, to understand emotional contagion, the analysis of physiological reactions should be combined with more sophisticated technology, of which neuroimaging tools offer many interesting possibilities.

### Emotional Contagion in Neurological Reactions

Emotional processing can be monitored using neurophysiological tools and neuroimaging tools, since the level of arousal can be associated with specific brain activity in the prefrontal cortex (Gray, 1970; Critchley et al., 2000; Dixon et al., 2017; Uhm et al., 2020). Neuroscience research has relied on neuroimaging tools for the study of basic emotions (Celeghin et al., 2017). For example, functional magnetic resonance imaging (fMRI) has been used for the study of basic emotions such as happiness, sadness, fear, anger, disgust, and surprise (Gu et al., 2019). Functional near-infrared spectroscopy (fNIRS) has been used to monitor the role of the prefrontal cortex in emotion processing (Doi et al., 2013). However, fNIRS uses relatively low spatial and temporal resolution. Electroencephalography (EEG) has been used to study affective states in the decision-making process (Li et al., 2020; Pei and Li, 2021). Due to the high temporal resolution of EEG, this neuroimaging tool has been widely employed to identify emotions, as an alternative to fNIRS or fMRI (Palmiero and Piccardi, 2017).

Previous neuroscience research in emotional contagion has inferred that various brain areas are associated with emotion processing (Papez, 1937; Panksepp, 1986; Hatfield et al., 1994; Harrison et al., 2006; Shamay-Tsoory, 2009; Dixon et al., 2017). In particular, positive and negative emotions are often identified located in the prefrontal cortex (Davidson et al., 1990; Lindquist et al., 2016; Xu et al., 2020). When subject to a combination of pleasure and arousal, these emotions reflect the core affect of emotional contagion (Hatfield et al., 1994). The prefrontal cortex also provides information about processing preferences (Boksem and Smidts, 2015; Casado-Aranda et al., 2019) or emotional processing of consumer behavior (Doi et al., 2013; Karmarkar and Yoon, 2016; Genevsky et al., 2017). Similarly, prefrontal cortex asymmetry has been widely employed to uncover consumers' decision-making process involving emotions (Davidson, 1988, Davidson et al., 1990; Harmon-Jones et al., 2010; Papousek et al., 2011; Ramsøy et al., 2018). Specifically, frontal EEG asymmetry is used to identify emotions during affective and cognitive processes (Grimshaw and Carmel, 2014; Palmiero and Piccardi, 2017).

Furthermore, neuroscience research has shown that the type of product, in terms of utilitarian or hedonic, also affects consumers' emotional responses (Bettiga et al., 2020). Since neuroimaging tools help to predict consumer behavior (Smidts et al., 2014; Cascio et al., 2015; Telpaz et al., 2015; Casado-Aranda et al., 2018a,b, 2020; Motoki et al., 2020; Jai et al., 2021), future lines of research should consider which environmental factors (such as types of products, types of purchase, etc.) impact and alter emotional responses, and ultimately, behavioral responses.

## Conclusions and Directions for Future Research

This study reviews and classifies the current research around emotional contagion, examining the theoretical backgrounds of the concept and identifying the various mechanisms and methodological approaches underpinning the processes of emotional contagion during human interactions. The theory of arousal (Mehrabian and Russell, 1974; Mehrabian, 1980; Russell, 1980, 2003) is the basis of the concept of emotional arousal, and explains how a combination of two elements—the level of pleasure (positive/negative) and the level of arousal (relaxed/activated)—trigger emotions. Emotional arousal is transmissible during human interactions, and this study reviews and classifies the methodological approaches and mechanisms that explain the contagious nature of emotions.

The literature review shows that academic research on emotional contagion has mainly focused on human interaction to understand the behavioral synchrony that results from emotional contagion. With the inclusion of artificial intelligence (AI) tools, such as chatbots or voice assistants, as a way of facilitating or supporting commercial interactions and transactions, future lines of research should also address the issue of emotional contagion in human–robot interactions (Matsui and Yamada, 2019). In particular, it would be interesting to extend the research on emotional contagion to study how the anthropomorphism level of AI can trigger different emotions in human–robot interactions.

Against this background, this study identified four different areas that describe the mechanism and effects of emotional contagion in human-to-human interactions. (1) Regarding emotional contagion in reaction to facial expressions, facial expressions can be cues of quality, customer-centricity, or attitudes that create positive or negative feelings to customers. In an online interaction when facial cues are not present, the tone and valence of voice can work as an emotional contagion agent. (2) Behavioral reactions can be a vehicle of emotional contagion. Such reactions can be expressed verbally or in written form; for example, in positive or negative customer reviews on online forums or social media. In an infamous experiment some years ago researchers manipulated and transformed people's emotions on a massive scale through social media posts (Kramer et al., 2014). (3) Physiological reactions, and (4) neurological reactions lead to emotional contagion. The emotional state of an agent can activate an autonomous nervous system response and neural representation of the affective state of the observer, and neurological reactions can be indicators of emotional contagion.

A number of neuroscience techniques and tools can be used to study the mental processes leading to emotional contagion. For example, EEG, fMRI, and fNIRS, as well as techniques such as eye-tracking, facial recognition, and skin conductance, offer advantages over traditional research techniques such as surveys or focus groups in studying the effects of emotional contagion. In particular, neuroscience tools can collect information on real-time and subconscious emotional reactions and behaviors, while traditional quantitative methods, such as self-reported techniques, merely record subjective experiences that are often biased (Bell et al., 2018; Verhulst et al., 2019; Uhm et al., 2020). Therefore, this integrated review points to the advantages of using neuroscience tools rather than traditional research tools to advance the understanding of how emotions are triggered in different sorts of interactions.

Future studies could advance the study of emotional contagion by comparing human interaction to human–robot interaction. Considering the increasing use of AI as a technology behind new tools based on anthropomorphism (voice assistants, chatbots, robots, etc.), in support of the marketing communication and transaction channels it is important that marketers are aware of the efficacy of such tools as agents of emotional contagion. However, marketers should also be aware of several ethical, and possibly even legal, implications of such techniques, which can also be subject to future research in this particular domain.

## Author's Note

This review tries to offer an overview of the concept of emotional contagion, through a literature review and exploration of future research directions.

## Author Contributions

All authors listed have made a substantial, direct and intellectual contribution to the work, and approved it for publication.

## Conflict of Interest

The authors declare that the research was conducted in the absence of any commercial or financial relationships that could be construed as a potential conflict of interest.
